# A three minutes supine position test reveals higher risk of spinal anesthesia induced hypotension during cesarean delivery. An observational study.

**DOI:** 10.12688/f1000research.15142.1

**Published:** 2018-07-09

**Authors:** Markos Erango, Arnoldo Frigessi, Leiv Arne Rosseland

**Affiliations:** 1 School of Mathematical and Statistical Sciences, Hawassa University, Hawassa, Ethiopia; 2 Oslo Centre for Biostatistics and Epidemiology, Oslo University Hospital, Oslo, Norway; 3 Department of Research and Development, Division of Emergencies and Critical Care, Oslo University Hospital, Oslo, Norway; 4 Institute of Clinical Medicine, Faculty of Medicine, University of Oslo, Oslo, Norway

**Keywords:** Blood pressure, spinal anesthesia, cardiac output, cesarean delivery

## Abstract

**Background:** Cesarean delivery is performed under spinal anesthesia, and vasodilation is the main cause for a drop in blood pressure. The compression of the aorta and inferior vena cava by the gravid uterus is of additional clinical importance. Hypotension may occur during cesarean delivery even if prophylactic infusion of phenylephrine is practiced. We have tested if a 3 minute supine observation, can identify a subset of women with decreasing systolic arterial pressure (SAP) under spinal anesthesia.

**Methods:** We performed a prospective observational study at Oslo University Hospital on healthy pregnant women for planned cesarean delivery. Continuous measurements of calibrated invasive SAP and estimated cardiac output were recorded for 76 women in a 3 minutes measurement with the woman in the left lateral position, followed by supine position for 3 minutes. Using functional data clustering, principal component analysis and curve smoothing, to filter way noise and reduce the dimensionality of the signal, we clustered the women into separate SAP groups.

**Results: **We identified two significantly different groups of women during supine position; one characterized by initial drop in SAP, the other showed initial increase. After spinal anesthesia, the mean SAP curve of the women in the first group showed a drop in blood pressure, which was more rapid than for the other women. A minor difference in cardiac output was observed between the two groups of women with the mean cardiac output curve for the first group being higher.

**Conclusions:** This work indicates that supine position affect clinically relevant cardiovascular measurements in pregnant women. A simple test may identify patients with increased risk of spinal anesthesia induced hypotension.

## List of abbreviations

SAP; systolic arterial pressure

Hb; hemoglobin concentration

BMI; body mass index

DAP; diastolic arterial pressure

MAP; mean arterial pressure

HR; heart rate

SV; stroke volume

CO; cardiac output

SVR; systemic vascular resistance

## Background

Spinal anesthesia is a standard method in cesarean deliveries and is regarded as being safe for the mother and the baby, even though significant maternal hypotension often occur ^1^. The vasodilatory effect of spinal anesthesia is the main cause for this drop in blood pressure ^2^. The compression of the aorta and inferior vena cava by the gravid uterus is known to reduce venous return and this is estimated to be of clinical importance after approximately 20 weeks of pregnancy. Pregnant women may find the supine position uncomfortable and avoid this position close to delivery. During cesarean delivery left lateral tilt is recommended to oppose this effect, and theoretically this should reduce the negative effects on maternal circulation. However, in clinical practice the incidence of spinal induced hypotension is high if no prophylactic measures to oppose the vasodilatation are included ^1^. We have previously compared two different prophylactic methods, lower leg compression and phenylephrine ^3^. In that study continuous infusion of phenylephrine was superior to lower leg compression and placebo. Lower leg compression was found to increase heart stroke volume compared to placebo indicating a significant effect on venous return. Some women have hypotension during cesarean delivery even if prophylactic infusion of phenylephrine and/or lower leg compression is practiced. Phenylephrine continuous infusion is not included as standard prophylaxis globally ^4^. Previous attempts to predict risk of spinal induced hypotension and to individualize the prophylactic treatment have not yet changed clinical practice.

### Aims

We tested if a 3 minutes supine observation prior to spinal anesthesia with continuous invasive hemodynamic measurements

a) Allows identification of a subset of women with decreasing blood pressure and/or cardiac output;

b) If this subset of women could be identified in left lateral positon;

c) If this subset of women showed any special properties in terms of cardiac output and systolic arterial pressure during cesarean delivery with phenylephrine prophylaxis under spinal anesthesia.

## Methods

The patients participated in a randomized, double-blinded, placebo-controlled, parallel-group comparison of carbetocin and oxytocin given intravenously during elective cesarean delivery under spinal anesthesia. The study was conducted at the Department of Anesthesiology, Division of Emergency and Critical Care, Oslo University Hospital, Rikshospitalet between November 2009 and September 2011. The Birth Clinic at Rikshospitalet is a tertiary care center, but the majority of the laboring women are healthy and representative of the general population in central Norway. The protocol was approved by the Data Inspectorate’s local representative at Oslo University Hospital, the Regional Committee for Medical and Health Research Ethics of Southern Norway (Oslo, Norway)(2009/130), and the Norwegian Medicines Agency (Oslo, Norway)(09/07301–7). This approval covered both the RCT and sub-study. The randomized controlled trial was registered at clinicaltrials.gov ( NCT00977769) and was conducted according to Good Clinical Trial practice, the CONSORT guidelines, and the principles of the Declaration of Helsinki. The results from the randomized controlled trial are published previously ^5^.

In addition to the RCT we performed an observational study. Detailed information about the observational study was integrated in the patient information and consent form. The observational study was conducted and reported according to STROBE guidelines ^6^.

Eligible participants were screened by the senior author (LAR) for inclusion at their last midwife consultation before their scheduled delivery. Oral and written information was given to each woman at least 24 hours before her delivery. Written informed consent was gained before inclusion. The women were informed about the intervention in the randomized controlled trial and the observational study being performed the last minutes before spinal anesthesia. They were asked to assess specifically the extra pain caused by to the arterial cannulation. The inclusion criteria included being in good health, a singleton pregnancy, being aged 18 years or older, and a scheduled planned cesarean section at a minimum gestational age of 36 weeks. The exclusion criteria included pre-eclampsia, placenta previa, placenta accreta, von Willebrand disease or other bleeding disorder, and systolic arterial pressure (SAP) < 90 mmHg. A total of 185 patients were screened, and 76 were included in the observational study. The indications for elective cesarean section were maternal request 43%, previous cesarean delivery 25%, breech presentation 8%, other obstetric reasons 13%, and other non-obstetric maternal or neonatal medical conditions 11%.

In addition to continuous beat-to-beat hemodynamic variables that were collected and stored electronically during left and supine position, we registered each patient’s age, height, weight, gestational age, preoperative hemoglobin concentration (Hb), hours fasting (liquids and solids), and the baby’s birth weight. High body mass index (BMI) may influence the hemodynamic response to both supine position and spinal anesthesia. Maternal weight upon inclusion was used to calculate the BMI (weight in kg / (height in meters) ^2^). An arterial catheter was placed into the radial artery after infiltration of lidocaine (10–20 mg) immediately after arrival at the operating theatre. Peripheral intravenous (i.v.) catheters were placed on both forearms. Invasive blood pressure were calibrated and continuous beat-to-beat measurements of invasive SAP, diastolic arterial pressure (DAP), mean arterial pressure (MAP), and heart rate (HR) were recorded in 3 minutes with the woman in a left lateral position. Stroke volume (SV), cardiac output (CO), systemic vascular resistance (SVR), and other estimated variables based on continuous arterial waveform analysis system were recorded using PulseCO (PulseCO ^TM^, Cambridge, United Kingdom), an integrated part of the LiDCO *plus* monitoring system (LiDCO Ltd., Cambridge, United Kingdom). We omitted the calibration of CO with the lithium dilution technique since the primary outcome was change in repeated measurements of hemodynamic variables. The hemodynamic observations were performed before spinal anesthesia was given. After the 3 minutes measurements in the left lateral position the women were turned into supine position for the 3 minutes test of aorto-caval compression. This experiment was not part of standard clinical procedure, and the women were encouraged to discontinue the test if they felt unwell.

With the woman in a right lateral position, spinal anesthesia was induced in the L2-L3 vertebral interspace, and bupivacaine 10 mg + fentanyl 20 µg was injected through a 25-G non-traumatic needle (Pencan®, B. Braun, Melsungen, Germany). Concomitantly, we started a rapid intravenous infusion of saline 0.9 mg/ml (37°C, 10 ml/kg) and a phenylephrine bolus (0.25 µg/kg). This was followed with a phenylephrine infusion (0.25 µg/kg/min). During surgery, the patient was supine with an operating wedge under her right hip (19° Tempur pillow). Hypotension (SAP< 90 mmHg) was treated with an extra i.v. bolus of phenylephrine 30 µg if the HR was above 60 beats per minute (bpm) or with i.v. ephedrine 5–10 mg if the HR was 60 bpm or below.

The hemodynamic data were stored prepared for statistical analysis according to methods described in detail previously ^5^. The data set used in the analyses of the supine test is open access available for analyses ( Dataset 1).

Phenylephrine prophylaxis (25 µg/kg/min i.v.) was started when spinal anesthesia was given. Due to the phenylephrine prophylaxis we expected only a minor decrease in SAP after induction of spinal anesthesia. The analyses of spinal induced hypotension were based on the beat-to-beat measurements during the first 5 minutes, guided by inspection of the blood pressure curves. Based on a previously published study we expected a maximum decrease in SAP 3–4 minutes after spinal anesthesia ^3, 7^.

### Statistical analyses

In order to capture the change of blood pressure, we considered the first order difference between two consecutive measurements *d _i_* ( *t*) = *b _i_* ( *t* + 1) – *b _i_* ( *t*), where *b _i_* ( *t*) is the average blood pressure of woman *i* measured in the 5 seconds before time *t*. The time *t* is discrete, so that *t* = 1, …, *T* = 40 corresponds to the three minutes measurement. Because data were still noisy, we smoothed the first order difference blood pressure curve *d _i_* ( *t*), *t* = 1, …, *T* of every woman, using a roughness penalty method ^8^, which led to a smoothed first order difference blood pressure curve denoted *s _i_* ( *t*), *t* = 1,…, *T* – 1. The method depends on a smoothing parameter *λ*. The results of the analysis below depend on the choice of *λ*. However, when clustering the women in separate groups, only a few women changed cluster when we changed the value of *λ*. To document such robustness, in the Supplementary material, we present results obtained for many values of *λ* ( Supplementary Table 1, Supplementary File 1 and Supplementary Figure 1). We found that λ=10000 allows to filter noise and capture details in the best way and in the rest we show the results for *λ=10000.*

We computed the principal components of the smoothed first order difference blood pressure curves *s _i_* ( *t*) for all women. The first two principal components represented in total 76.9% of the total variation of the data and were used for clustering. We clustered the 76 women using these two first principal components, into two or three groups. We used the k-means method ^9^, implemented in *cclust* package (Version 0.6–21) ^10^. More details and an intuitive description of the statistical methods are given in the Supplementary material ( Supplementary File 1).

We used the multivariate paired Hotelling’s T ^2^ test ^11^ to test the null hypothesis that the mean curves of the two clusters are equal for all time points against the alternative that the mean vectors of the two groups are not equal for all time points (H _0_: **μ**_1_ = **μ**_2_ vs. H _1_: **μ**_1_≠ **μ**_2_) where ***μ***_1_ is the mean curve vector for S1 and ***μ***_2_ is the mean curve vector for S2. For comparison of the three clusters, we used the multivariate MANOVA to test the null hypothesis that the mean curves of are equal for all time points against the alternative that the mean vectors of the three groups are not equal for all time points (H _0_: **μ**_1_ = **μ**_2_ = **μ**_3_ vs. H _1_: **μ**_1_≠ **μ**_2_ ≠ **μ**_3_), where ***μ***_1_ is the mean curve vector for T1, ***μ***_2_ is the mean curve vector for T2 and ***μ***_3_ is the mean curve vector for T3. Statistical analyses were performed with R-software versions 3.2 to 3.3.2.

## Results

Baseline measurements of HR, SAP, MAP, DAP, BMI, age, parity, gestational age, hours in fasting for solids, and estimated intake of liquids for the 76 included women are presented in Table 1. Mean supine SAP curves of the women with increasing values are available as Supplementary Figures 2. The principle component analysis identified two groups of women, by clustering the smoothed blood pressure difference curves during supine position into two groups as described in the methods. The first group, denoted S1, included 30 women, the second group, S2, the remaining 46 women. There were no statistically significant differences between the two clusters in baseline characteristics (see Supplementary Table 2).

**Table 1.** Patient characteristics.

Age (years)	34.5 (21, 43)
BMI (kg/m ^2^)	28.5 (SD 4.3)
Nulliparous	17 (of 76)
GA (weeks)	39 (36, 40)
Preop Hb (g/100 mL)	11.3 (SD 1.1)
Preop fasting (h)	12.0 (SD 2.8)
Baseline ^a^:	
Syst AP (mmHg)	134 (SD 14.0)
Mean AP (mmHg)	91 (SD 9.2)
Diast AP (mmHg)	68 (SD 7.5)
HR (bpm)	77 (SD 11.6)

Data presented as mean (SD, range, or total number). BMI; body mass index, GA; gestational age, a; baseline values representing a 60 sec mean of intra-arterial blood pressure (AP), and heart rate (HR) with the patient in left lateral position.

**Figure 1.**
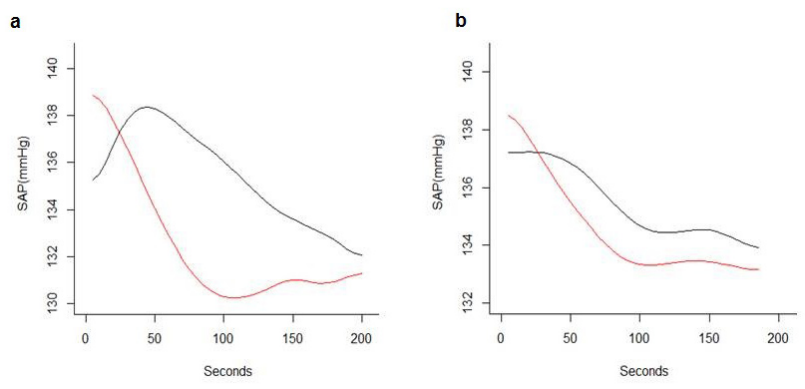
Mean smoothed systolic arterial pressure (SAP) curves for S1 and S2 during supine position (mmHg) (panel **a**) and mean smoothed SAP curves for S1 and S2 during left lateral position (mmHg) (panel **b**). S1 in red, S2 in black.

Figure 1 shows the mean SAP curves for the two groups, which are characterized by different trajectories. During supine position the mean SAP of the S2 patients dropped by about 10 mmHg in the first 100 seconds. After that the blood pressure was roughly constant. In the S1 group, the estimated mean SAP increased in the first 50 seconds of about 5 mmHg, and then started to decrease. The decrease was however less sharp than for group S2 (T2 = 216.89, p-value <0.001). The raw data curves of SAP for S1 and S2 groups are presented as Supplementary Figure 3.

We looked further into possible characterizations of the groups S1 and S2. We computed the standard deviation (SD) of the two groups for each time point. We found that S1 had minimum variation after 35 seconds, and then from the 100th seconds onwards had stable SD, whereas the SD in group S2 had a maximum at start and a minimum after 85 seconds (see Supplementary Figure 4).

In a second analysis, we clustered the 76 women into *three* groups. The reason for this was that the percentage (60%) of women in S2, characterized by a mean blood pressure drop, appeared to be larger than expected from clinical experience ^12^. When repeating the same analysis with three groups, we found a first group T1 with 28 women (36.8%) and a second group T2 with 15 women (19.7%) for which the average blood pressure increased in the first 30–50 seconds before then starting to drop. The difference between these two groups was in the different rate of decay, stronger for the women in T2. The third group T3 (43.5%) was characterized by a sharp drop in the average blood pressure right away, of approximately 10 mmHg in the first 100 seconds. After that the mean blood pressure was roughly constant. The three groups are not equal (Wilks Lambda= 0.033481, p-value<0.001). Mean hemodynamic measurements in left lateral and supine position defined by the principle components are presented in Supplementary Table 3.

### Comparison between supine and left lateral position

**Figure 2.**
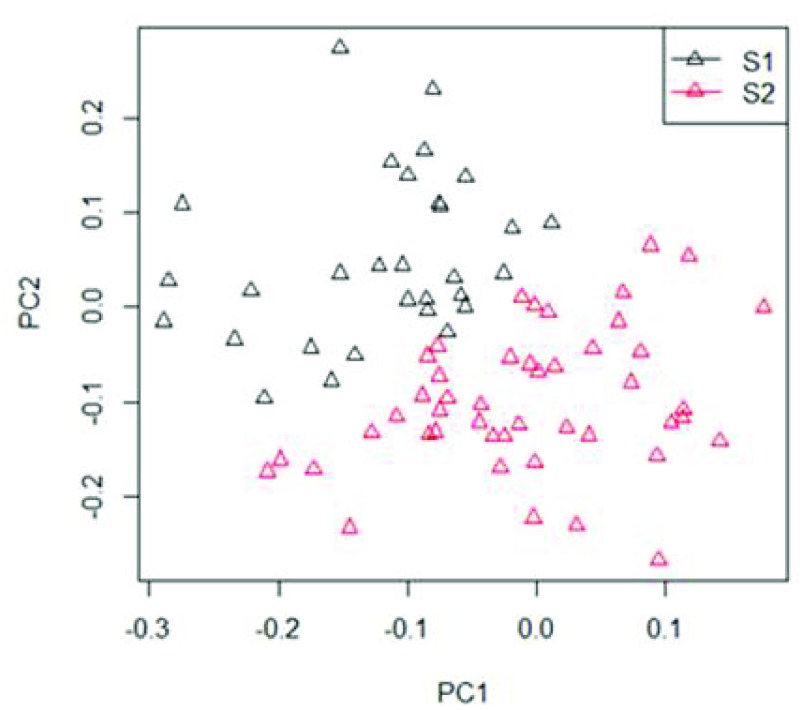
Principal component analysis of continuously measured systolic arterial pressure (SAP) from 76 women in supine position into two groups S1 (black) and S2 (red). There is one point per woman, with coordinates given with respect to the first and second principal component. PC= principal component.

We investigated if the clusters defined on the basis of the measurements while in supine position could be associated with blood pressure patterns while on left lateral position. This was not the case, as the mean blood pressures while on left lateral position for groups S1 and S2 were not significantly different ( Figure 1). The present data indicated that the drop in blood pressure when put in supine position for one group of women (S2), was not reflected in a blood pressure pattern when on the side which was different from the women in S1. Figure 2 shows how the first two principal components allow clustering the women into two groups (S1 and S2). The clustering algorithm puts each woman in one of two groups, so to divide them in the best way. The two clusters are nicely distinct, though there are borderline women.

### Comparison between supine position and during spinal anesthesia

**Figure 3.**
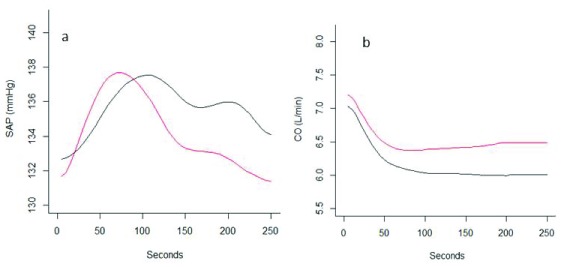
Mean systolic arterial pressure (SAP)(mmHg) (panel **a**) and cardiac output (CO)(L/min) (panel **b**) after spinal anesthesia in the two clusters (S1 and S2).

When comparing the two clusters (S1 and S2) in terms of their mean SAP curves, we found that for all women in both groups mean SAP increased roughly 8 mmHg in the first 80–100 seconds after spinal anesthesia. After that, mean SAP started to decrease in both groups (S1 and S2), but the blood pressure drop for the women in S2 was clearly sharper than for the women in S1 ( Figure 3). We found that the group of women who experience a drop of blood pressure when put in supine position (S2) also have a drop which is faster and towards lower values compared to the women (in S1) who in supine position have a non-dropping blood pressure (S1) ( Figure 3a). Mean cardiac output decreased in both groups but more in the group S1 than in S2 ( Figure 3b). The mean SAP curves during anesthesia of the two groups were different (T2 = 370.5, p-value <0.001) as were CO (T2=294.3, p-value <0.001). Due to hypotension (SAP< 90 mmHg) three group S2 patients were given phenylephrine bolus 30 µg. None in group S1 received extra bolus phenylephrine.

When we considered SAP *difference* under spinal anesthesia (SAP (time=250 sec) – SAP (time=10 sec)) compared with SAP *difference* during the supine period (SAP (time=180 sec) – SAP (time=10 sec)), the correlation in S1 was -0.158 (p-value =0.40) and the correlation in S2 -0.48 (p-value = 0.0008). When we consider SAP difference under spinal anesthesia in the period from 90 sec (peak SAP, see Figure 3a) and 250 sec (SAP (time=250 sec) – SAP (time=90 sec)) compared with SAP during supine (SAP (time=180 sec) – SAP (time=10 sec)), the correlation in S1 was 0.095 (p-value = 0.62) the correlation in S2 was 0.15 (p-value = 0.32).

**Figure 4.**
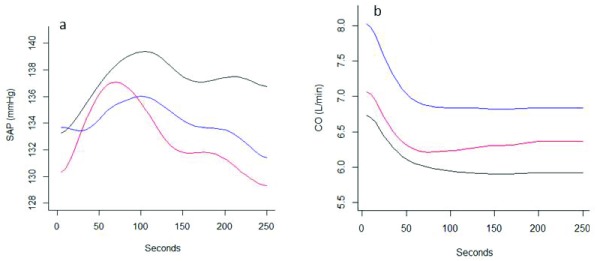
Mean systolic arterial pressure (SAP)(mmHg) and CO (L/min) after spinal anesthesia in the three clusters T1, T2, and T3.1.

When repeating this analysis with the three groups division (T1, T2 and T3) we observed a comparable pattern ( Figure 4). Mean SAP increased during the initial 50–100 seconds followed by a decrease which was different for the three groups. The group of women with the most prominent drop in blood pressure when put in supine position (T3), also had the largest drop in average blood pressure under spinal anesthesia, and was still falling after 250 seconds. The group of women with the smallest drop in blood pressure in supine position (T1) did not have any drop in blood pressure under spinal anesthesia, with mean SAP remained constant. The intermediate group of women (T2), with some drop of blood pressure when in supine position, also had some drop under spinal anesthesia, but it was a slower drop than what was observed for T3 ( Figure 4a). Mean cardiac output decreased in all three groups ( Figure 4b). The group differences in mean SAP and CO curves for all time points were statistically significant (p-value < 0.001)(MANOVA test, Wilks’ *Lambda*= 0.31862, F-value= 31.36, and Wilks’ *Lambda* = 0.17622, F-value= 48.307, respectively).

Complete continuous invasive hemodynamic measurements in both left lateral and supine positions Click here for additional data file. Copyright: © 2018 Erango M et al. 2018 Data associated with the article are available under the terms of the Creative Commons Zero "No rights reserved" data waiver (CC0 1.0 Public domain dedication).

## Discussion

The three minutes supine observation test prior to spinal anesthesia identified women with risk of spinal induced hypotension. This finding is in concordance with previous studies reporting an association between heart rate increase or hypotension in the supine position and subsequent development of spinal hypotension and/or vasopressor requirements ^13– 15^. Kinsella *et al*., studied non-invasive arterial pressure and heart rate during a 5 min supine position test ^13^, and concluded that it was not possible to identify women with increased risk of spinal induced hypotension based on the observation of changes in arterial pressure. In contrast, the patients with a heart rate increase above 10 beats/min were more likely to develop hypotension. The novelty of our method is the potential benefit of in the future being able to predict hypotension and thereby individualize prophylactic therapy. We also demonstrated that this physiologic phenomenon present in the supine position was absent when observing the same women in left lateral position, supporting the concept of aorto-caval compression as the dominant mechanism of hypotension in the supine position ^13^.

 The cluster of supine women having a falling SAP trend developed lower mean SAP and higher mean CO after spinal anesthesia. The decrease in systemic vascular resistance following spinal anesthesia is the dominant mechanism of spinal hypotension ^2^. A statistically significant difference in mean CO was observed between the two groups of women and the group with lowest SAP had a higher CO. The difference in CO was probably of limited clinical significance but indicates that the difference in the compensation of spinal anesthesia induced vasodilatation is the mechanism behind this observation. Women are more dependent on sympathetic tone during pregnancy than in non-pregnant state and the effect of spinal anesthesia is a rapid and prominent vasodilatation, leading to a decrease in blood pressure and increase in CO ^7, 16^. All women in this study received standard phenylephrine bolus and infusion, and this prophylaxis was effective as the mean SAP was stable ( Supplementary Figure 2c). However, three women had a drop in SAP below 90 mmHg and needed extra doses of phenylephrine to keep SAP close to baseline. All three women belonged to the group characterized with drop in SAP during supine position.

It was evident that the principal component analysis could not be replaced by physician observation and visual assessment of the continuous SAP curves, as the variance is large and individual changes difficult to interpret ( Supplementary Figure 2a). Functional data analysis with principal component analysis of continuous invasive measurements of SAP made a distinction between two separate clusters, one of the two characterized by decreasing mean SAP. It is important to notice that the two groups are not created because the women in S1 started with a lower average blood pressure than the women in S2. The two groups were created from the first order difference blood pressures, where the level does not have any influence. Principal component analyses have been used for making predictive models. In this population it would be interesting to identify patients at risk of severe spinal anesthesia induced hypotension and tailor the prophylactic treatment. The reliability of this method has to be proven in a new independent study.

Heart rate variability analysis captures the autonomic nervous activity and has been shown to predict spinal anesthesia induced hypotension in pregnant women ^17, 18^, and the ratio of the low frequency and the high frequency bands (LF/HF) correlated with spinal induced hypotension. Spinal anesthesia induced hypotension is caused by the vasodilatory effects, and to a lesser extent by aorto-caval compression ^5^, and the relationship between estimated autonomic activity (LF/HF) and spinal anesthesia induced hypotension is likely. The requirements of heart rate variability analyses limits the feasibility as stable ECG-signals has to be monitored at least 5 minutes to perform the frequency domain analyses ^19^. Sakata *et al.,* analyzed heart rate variability during postural changes and found a correlation between low-to-high frequency ratio shift and spinal induced hypotension during cesarean delivery ^18^. Time domain analyses or continuous wavelet transform may be applicable ^20^ and requires shorter heart rate recordings. However, the method is not yet validated in the obstetric population.

Pulse transit time measures the interval from the electrical activity to the physical pulse wave in the periphery. Pulse transit time provide rapidly available beat-to-beat information of changing hemodynamics and may estimate the real time vasodilatory effect of spinal anesthesia ^21^, and is not suitable in a prediction model. 

The external validity of a classification algorithm based on functional data analysis and clustering, as suggested in this paper, needs to be investigated in similar clinical assays. In this study all patients were treated according to a protocol including phenylephrine starting bolus and continuous infusion and we expected the blood pressure to be quite stable. Treatment protocols without intravenous phenylephrine prophylaxis will be different; Hypotension will be more profound and occur over a longer time sequence ^3^.

In a randomized placebo-controlled trial we showed that lower leg wrapping was less effective than phenylephrine bolus + infusion regarding spinal induced hypotension ^3^. Cardiac output increased in all groups. The study confirmed that spinal anesthesia is vasodilatory and that the changes in venous return, represented by heart stroke volume, were minor. Interestingly, heart stroke volume was higher in the lower leg wrapping group indicating some effect on venous return. Individual differences in the ability to compensate a fall in venous return may be the mechanism observed in the three minute supine test. Even though vasodilation is the main effect of spinal anesthesia it would be of interest to study how the prediction model works together with mechanical lower leg wrapping or when no hypotension prophylaxis is administered.

The algorithm should also be evaluated using a non-invasive continuous monitoring device. Non-invasive, continuous, beat-to-beat monitoring of blood pressure is available and included in clinical practice ^22^ and invasive monitoring is not standard clinical practice in obstetric anesthesia. Two classes of devices have been developed; Tonometric devices and volume clamp devices. The limits-of-agreement analyses of the latter 2 device classes using invasive measures as a reference standard are promising ^22^. Non-invasive measurement of blood pressure is not yet validated in obstetric anesthesia.

## Conclusions

This work indicates the possibility to identify patients with increased risk of spinal anesthesia induced hypotension based on a simple pre-anesthesia test.

## Data availability

The data referenced by this article are under copyright with the following copyright statement: Copyright: © 2018 Erango M et al.

Data associated with the article are available under the terms of the Creative Commons Zero "No rights reserved" data waiver (CC0 1.0 Public domain dedication). 

Dataset 1: Complete continuous invasive hemodynamic measurements in both left lateral and supine positions 10.5256/f1000research.15142.d209363 ^23^

## Supplementary material

**Supplementary File 1- File containing expansion of the statistical methods and the following supplementary tables:**

Supplementary Table 1: Baseline demographics in the cluster S1 and S2.

Supplementary Table 2: Assignment of each woman to one of the two groups for various values of λ

**Supplementary File 2: File containing the following supplementary table:**

Supplementary Table 3: Hemodynamic variables in clusters defined by principle components

**Supplementary Figure 1: plot for each of the values of λ which were tested, the mean supine SAP of the women in each of the two groups**. This is the mean of the smoothed supine SAP curves of each woman, and therefore depends on λ. With increasing values of λ, the two curves become more regular. There is little change, but noise is progressively reduced. By smoothing the data, noise is filtered out allowing for a better signal. The important observation is that the results are very stable for λ larger than 5000. This confirms that the choice of λ=10000 is appropriate, and that results would be qualitatively unchanged for other values of λ around 10000.

**Supplementary Figure 2: SAP of all women during lateral position (a), supine position (b) and during anesthesia (c), and (in bold red) average SAP at each time point**. We did not notice any general clear trend, though the average SAP had a weak negative decay. Notice the large variance among the women.

**Supplementary Figure 3: Smoothed SAP curves for S1 and S2 during supine position and mean SAP (mmHg)**. Notice the large variability. The scale of the y-axis is much larger than in Figure 2 in the paper, to permit the plot of all women data.

**Supplementary Figure 4: Standard deviation (SD) of the smoothed SAP curves for S1 and S2 during three minutes supine position, time point per time point.** There was a high variability in each group, clearly more in the S2 group. We therefore tried with three groups, so that variance in each groups will be less. Notice that the principle component analysis allows filtering out noise.

## References

[1] Cyna AM Andrew M Emmett RS: Techniques for preventing hypotension during spinal anaesthesia for caesarean section. *Cochrane Database Syst Rev.* 2006; (4):Cd002251. 10.1002/14651858.CD002251.pub2 17054153

[2] Assali NS Prystowsky H: Studies on autonomic blockade. I. Comparison between the effects of tetraethylammonium chloride (TEAC) and high selective spinal anesthesia on blood pressure of normal and toxemic pregnancy. *J Clin Invest.* 1950;29(10):1354–1366. 10.1172/JCI102373 14778899 PMC436179

[3] Kuhn JC Hauge TH Rosseland LA: Hemodynamics of Phenylephrine Infusion Versus Lower Extremity Compression During Spinal Anesthesia for Cesarean Delivery: A Randomized, Double-Blind, Placebo-Controlled Study. * Anesth Analg.* 2016;122(4):1120–1129. 10.1213/ANE.0000000000001174 26991619

[4] Ngan Kee WD: The use of vasopressors during spinal anaesthesia for caesarean section. *Curr Opin Anaesthesiol.* 2017;30(3):319–325. 10.1097/ACO.0000000000000453 28277383

[5] Rosseland LA Hauge TH Grindheim G: Changes in blood pressure and cardiac output during cesarean delivery: the effects of oxytocin and carbetocin compared with placebo. *Anesthesiology.* 2013;119(3):541–551. 10.1097/ALN.0b013e31829416dd 23598289

[6] von Elm E Altman DG Egger M: The Strengthening the Reporting of Observational Studies in Epidemiology (STROBE) statement: guidelines for reporting observational studies. * Lancet.* 2007;370(9596):1453–1457. 10.1016/S0140-6736(07)61602-X 18064739

[7] Langesaeter E Rosseland LA Stubhaug A: Continuous invasive blood pressure and cardiac output monitoring during cesarean delivery: a randomized, double-blind comparison of low-dose versus high-dose spinal anesthesia with intravenous phenylephrine or placebo infusion. *Anesthesiology.* 2008;109(5):856–863. 10.1097/ALN.0b013e31818a401f 18946298

[8] Ramsay J Silvermann B: Functional Data Analysis. Springer Series in Statistics.In: Wiley Online Library,1998.

[9] Kanungo T Mount DM Netanyahu NS: An efficient k-means clustering algorithm: Analysis and implementation. *IEEE transactions on pattern analysis and machine intelligence.* 2002;24:881–892. 10.1109/TPAMI.2002.1017616

[10] Dimitriadou E Hornik K Hornik MK: Package ‘cclust’.2015 Reference Source

[11] Anderson T: An Introduction to Multivariate Analysis.John Wiley & Sons (New York).2003 Reference Source

[12] Kinsella SM Lohmann G: Supine hypotensive syndrome. *Obstet Gynecol.* 1994;83(5 Pt 1):774–788. 8164943

[13] Kinsella SM Norris MC: Advance prediction of hypotension at cesarean delivery under spinal anesthesia. *Int J Obstet Anesth.* 1996;5(1):3–7. 10.1016/S0959-289X(96)80067-7 15321375

[14] Dahlgren G Granath F Wessel H: Prediction of hypotension during spinal anesthesia for Cesarean section and its relation to the effect of crystalloid or colloid preload. *Int J Obstet Anesth.* 2007;16(2):128–134. 10.1016/j.ijoa.2006.10.006 17276668

[15] Jeon YT Hwang JW Kim MH: Positional blood pressure change and the risk of hypotension during spinal anesthesia for cesarean delivery: an observational study. *Anesth Analg.* 2010;111(3):712–715. 10.1213/ANE.0b013e3181e8137b 20686012

[16] Sharwood-Smith G Drummond GB: Hypotension in obstetric spinal anaesthesia: a lesson from pre-eclampsia. *Br J Anaesth.* 2009;102(3):291–294. 10.1093/bja/aep003 19218369

[17] Hanss R Bein B Francksen H: Heart rate variability-guided prophylactic treatment of severe hypotension after subarachnoid block for elective cesarean delivery. *Anesthesiology.* 2006;104(4):635–643. 16571956 10.1097/00000542-200604000-00005

[18] Sakata K Yoshimura N Tanabe K: Prediction of hypotension during spinal anesthesia for elective cesarean section by altered heart rate variability induced by postural change. *Int J Obstet Anesth.* 2017;29:34–38. 10.1016/j.ijoa.2016.09.004 27789074

[19] Heart rate variability: standards of measurement, physiological interpretation and clinical use. Task Force of the European Society of Cardiology and the North American Society of Pacing and Electrophysiology. *Circulation.* 1996;93(5):1043–1065. 10.1161/01.CIR.93.5.1043 8598068

[20] Wachowiak M Hay D Johnson M: Quantification of Wavelet Band Metrics for Assessing Heart Rate Variability.In: *World Congress on Medical Physics and Biomedical Engineering, June 7-12, 2015, Toronto, Canada* Springer,2015:1026–1029. 10.1007/978-3-319-19387-8_250

[21] Sharwood-Smith G Bruce J Drummond G: Assessment of pulse transit time to indicate cardiovascular changes during obstetric spinal anaesthesia. *Br J Anaesth.* 2006;96(1):100–105. 10.1093/bja/aei266 16257996

[22] Bartels K Esper SA Thiele RH: Blood Pressure Monitoring for the Anesthesiologist: A Practical Review. *Anesth Analg.* 2016;122(6):1866–1879. 10.1213/ANE.0000000000001340 27195632

[23] Erango M Frigessi A Rosseland LA: Dataset 1 in: A three minutes supine position test reveals higher risk of spinal anesthesia induced hypotension during cesarean delivery. An observational study. *F1000Research.* 2018 10.5256/f1000research.15142.d209363 PMC6085602 30135733

