# Supplementary material for: A three minutes supine position test reveals higher risk of spinal anesthesia induced hypotension during cesarean delivery. An observational study
Source: F1000Res. 2018 Jul 9;7:1028. [Version 1] doi: 10.12688/f1000research.15142.1 (PMC6085602; doi:10.12688/f1000research.15142.1)
Supplement: Supplementary file 3 [file f1000research-7-16496-s0002.tgz › aba1c95d-445d-4f33-81c6-870eac1dc3b6.docx]

*Supplementary material file 2*

A three minutes supine position test reveals higher risk of spinal anesthesia induced hypotension during cesarean delivery. An observational study.

Supplementary table 3

**Hemodynamic variables in clusters defined by principle components**

Two clusters S1 and S2

**Cluster S1 left S2 left S1 supine S2 supine**

**SAP**  133.2 (14.5) 131.9 (13.2) 135.4 (12.8) 132.6 (14.0)

**DAP** 68.0 (7.9) 68.2 (6.9) 68.2 (7.7) 66.7 (6.4)

**MAP** 90.4 (9.2) 90.6 (8.7) 92.3 (9.1) 90.5 (8.2)

**HR** 75.8 (12.0) 78.8 (11.2) 75.9 (11.2) 79.6 (11.1)

**SV** 85.2 (7.8) 85.9 (8.8) 80.2 (7.8) 81.3 (10.6)

**CO** 6.4 (1.1) 6.7 (1.2) 6.1 (1.0) 6.6 (1.3)

**SVR** 1083 (233) 1036 (227) 1112 (240) 1028 (230)

Three clusters T1, T2, and T3

**Cluster T1 left T2 left T3 left T1 supine T2 supine T3 supine**

**SAP** 134.9 (14.2) 132.6 (15.8) 130.3 (12.2) 137.2 (12.4) 134.0 (15.6) 130.6 (13.1)

**DAP** 70.0 (7.1) 64.6 (7.2) 68.2 (7.0) 69.8 (6.8) 63.8 (6.2) 66.7 (6.8)

**MAP** 93.3 (8.8) 86.4 (8.0) 90.0 (8.7) 94.6 (8.7) 87.4 (6.8) 90.0 (8.3)

**HR** 73.7 (8.7) 82.9 (13.2) 78.6 (12.0) 74.0 (9.0) 82.3 (12.8) 79.9 (11.4)

**SV** 84.8 (6.0) 88.7 (9.0) 84.9 (8.7) 81.4 (9.4) 83.9 (8.5) 79.1 (9.9)

**CO** 6.3 (0.9) 7.1 (1.3) 6.5 (1.2) 6.1 (0.8) 7.0 (1.3) 6.4 (1.3)

**SVR** 1026 (211) 918 (187) 1155 (238) 1150 (220) 919 (170) 1050 (246)

Hemodynamic measurements during left lateral and supine position presented as mean (standard deviation = SD) of the individual woman’s two minutes mean beat-to-beat measurements. SAP; systolic arterial pressure (mmHg), DAP; diastolic arterial pressure (mmHg), MAP; mean arterial pressure (mmHg), HR; heart rate (beats per minute), SV; cardiac stroke volume (mL), CO; cardiac output (L/minute), SVR; systemic vascular resistance (dynes •s/cm^5^).
