# Supplementary figures and images for: A three minutes supine position test reveals higher risk of spinal anesthesia induced hypotension during cesarean delivery. An observational study
Source: F1000Res. 2018 Jul 9;7:1028. [Version 1] doi: 10.12688/f1000research.15142.1 (PMC6085602; doi:10.12688/f1000research.15142.1)

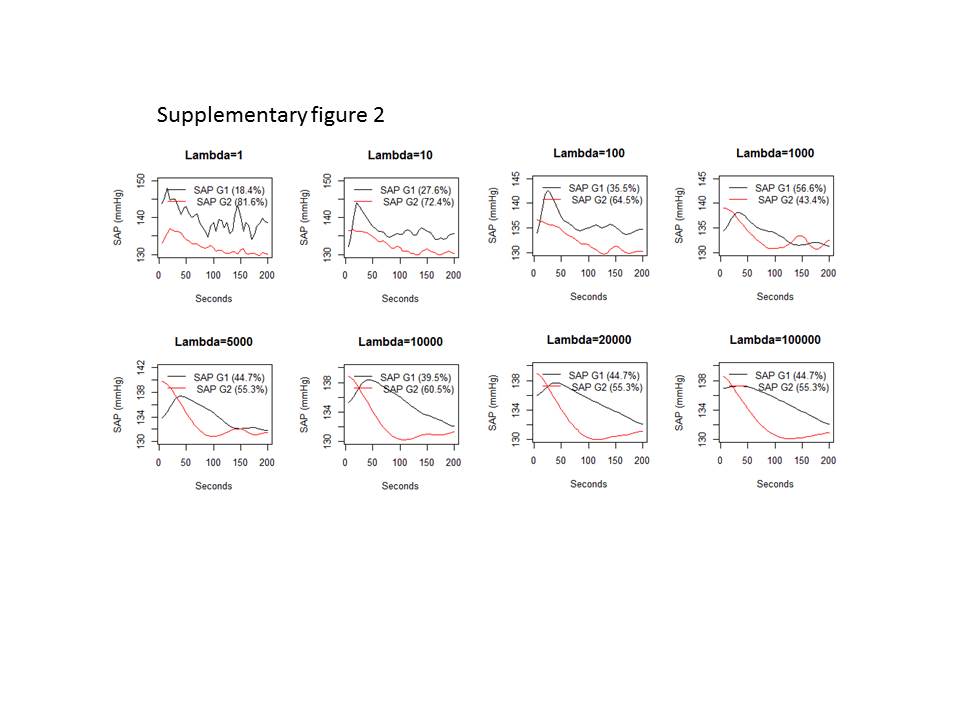

Supplement: Supplementary file 4 [file f1000research-7-16496-s0003.tgz › ce6c5d50-8c3b-4480-ac2c-ee19d4fe6efe.jpg]

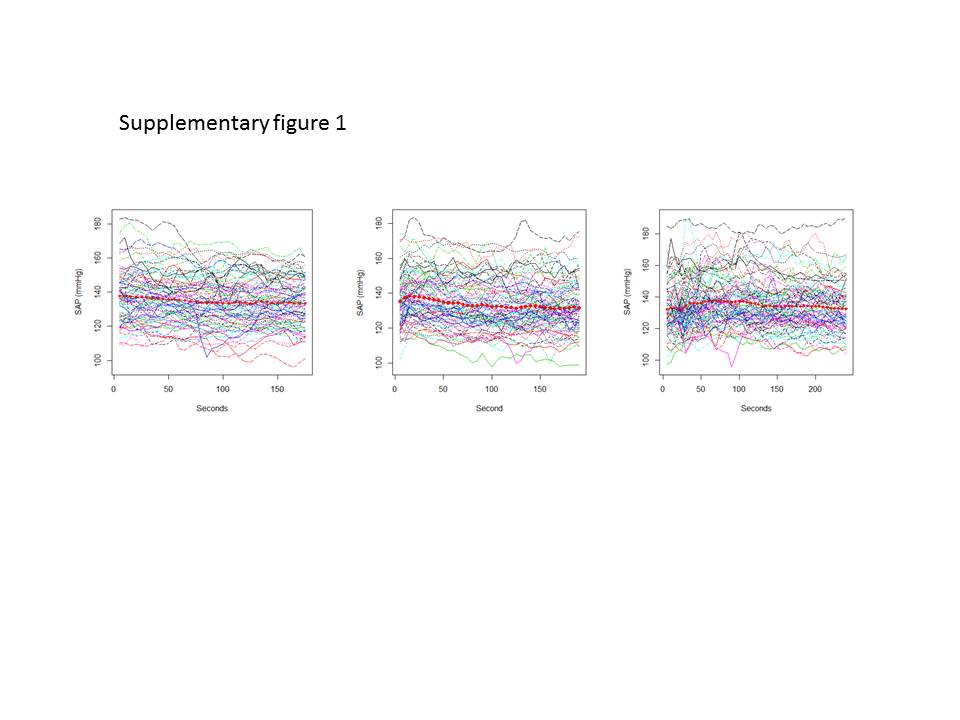

Supplement: Supplementary file 5 [file f1000research-7-16496-s0004.tgz › 88260a14-f69f-4945-82b4-348fe106e2e5.jpg]

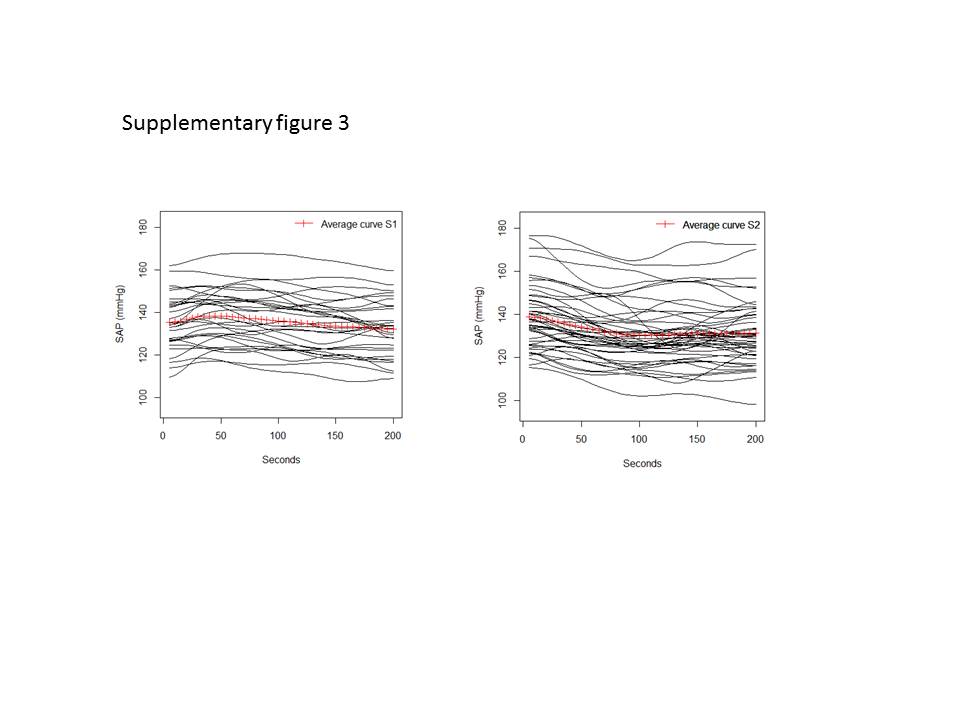

Supplement: Supplementary file 6 [file f1000research-7-16496-s0005.tgz › 0490f93e-608a-4ff8-9bb9-59343996bd0c.jpg]

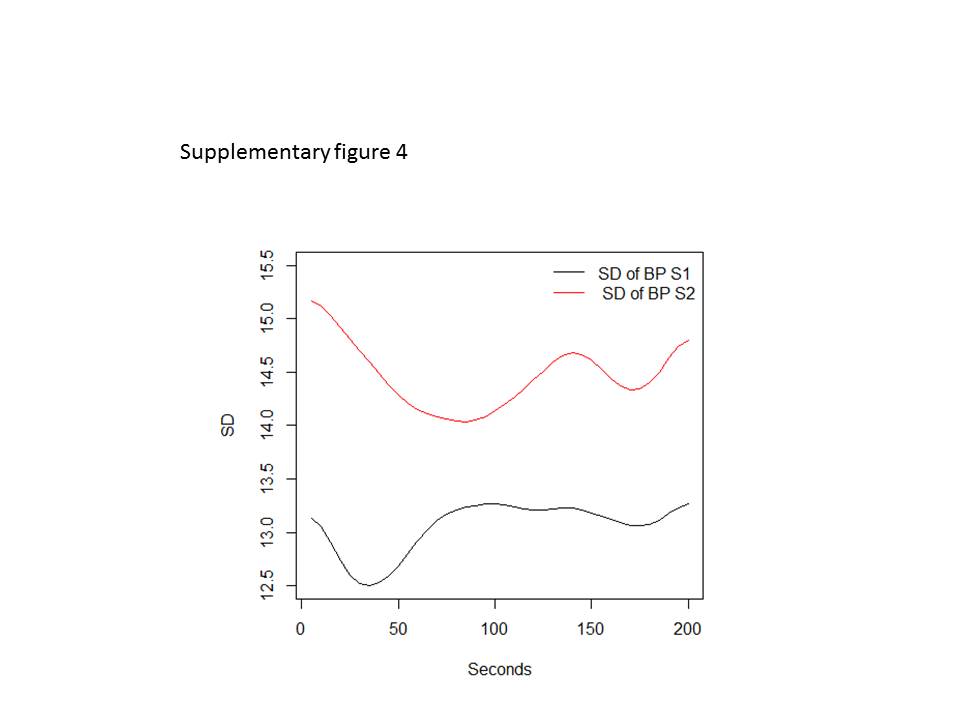

Supplement: Supplementary file 7 [file f1000research-7-16496-s0006.tgz › ba1da5f2-c022-4264-9968-f31f632fa299.jpg]
