# Supplementary material for: A three minutes supine position test reveals higher risk of spinal anesthesia induced hypotension during cesarean delivery. An observational study
Source: F1000Res. 2018 Jul 9;7:1028. [Version 1] doi: 10.12688/f1000research.15142.1 (PMC6085602; doi:10.12688/f1000research.15142.1)
Supplement: Supplementary file 2 [file f1000research-7-16496-s0001.tgz › 730a7662-fd0d-448f-a97d-c8cea241f9d3.doc]

Supplementary materials file 1

A three minutes supine position test reveals higher risk of spinal anesthesia induced hypotension during cesarean delivery. An observational study.

**1. Raw data**

Individual beat-to-beat measurements of systolic arterial pressure are presented as plot of raw data (Supplementary figure 1).

**2. Statistical analyses**

We computed the principal components of the smoothed first order difference blood pressure curves for all women, See Jolliffe, I. Principal component analysis (Joliffe, 2002).

Let X be the data matrix, composed with as many rows as women, and as many columns as measurements, here 38. The matrix then has the SAP of woman i at time point j in position (i,j). Principal component Analysis (PCA) is a method to decompose the data matrix X into a number of components t, which are called principal components, given as linear combinations of the original variables

$t_{k}=p_{k1}X_{1}+...+p_{kp}X_{p}$,

k = 1, …., p, where the weights $p_{k1},....,p_{kp}$constitutes the k-th eigenvector of the covariance matrix of X. Every woman has a vector of p scores, while the weights are in common to all women. The idea now is that the first F principal components will capture most of the information in X. One can show that the percent variation explained by the first F principal components is the sum of the F corresponding eigenvalues. For example, for the data set of supine SAP, the percent variation explained by the first two components is

,

where p is the number of measurement, here 38. In this case the first two principal components explain 77% of the total variation seen in the data in Supplementary figure 1b. If we would use also the next and third principal component, we would explain 18% more, and so on, indicating that stopping with 2 is a good balance between approximation and parsimony.

The first two principal components were used for clustering: every woman is characterized by two numbers, one related to the first and the other to the second principal component. In this way we have reduced the data from the matrix X with 38 columns to a matrix with only 2 columns. Now imagine that we plot these two numbers as coordinates in the plane. Every woman is a dot in this plane. We will see that some women are very close to each other, other are more far apart. Clustering in two groups means to divide the 76 women into two groups so that the distance of the corresponding dots of the women in each group is small compared to the distance between women belonging each to one of the two groups. There are many ways to compute such two groups (or clusters). We used the k-means method, implemented in cclust package (Dimitriadou, 2015). The algorithm moves women between the two clusters, trying to minimize the within cluster distance while maximizing the between cluster distance. We used the standard Euclidean distance in this paper.

**3. Robustness of results with respect to the smoothing parameter λ**

When we smooth the curves of supine SAP with spline smoothing, we have to decide about the value of the smoothing parameter λ. If λ is small, there is no smoothing. If it is large, the curve of each woman is smoother and in the limit is constant all along. For a series of selected values of λ, we show the two chosen clusters. In the second column, we have used λ=1, and the first women is in group 2, while women 12 is in group 1. The next column is for λ=10 and so on, for increasing values of λ. We see that the results become to be stable for larger values of λ. Robustness in the clusters is achieved if the women do not change group anymore, for consecutive increasing values of λ. Some women are always in the same group, others are first in one group and then stably in the other. Some women are more borderline between the two groups and fluctuate a bit (like women 3). We conclude that results are robust especially for values of λ larger or equal to 10.000. We report the results for λ=10000 in the paper (Supplementary table 2).

Supplementary Table 1

Assignment of each woman to one of the two groups for various values of λ

|  | **Lambda** | | | | | | | |
| --- | --- | --- | --- | --- | --- | --- | --- | --- |
| **Women** | 1 | 10 | 100 | 1000 | 5000 | 10000 | 20000 | 10000 |
| 1 | **2** | **2** | **2** | **2** | **2** | **2** | **2** | **2** |
| 2 | **2** | **2** | **1** | **2** | **2** | **2** | **2** | **2** |
| 3 | **2** | **1** | **1** | **1** | **2** | **2** | **1** | **1** |
| 4 | **2** | **2** | **2** | **2** | **2** | **2** | **2** | **1** |
| 5 | **2** | **2** | **2** | **2** | **2** | **2** | **2** | **2** |
| 6 | **2** | **2** | **2** | **1** | **1** | **1** | **1** | **1** |
| 7 | **2** | **2** | **2** | **1** | **1** | **1** | **1** | **1** |
| 8 | **2** | **2** | **2** | **1** | **1** | **2** | **2** | **2** |
| 9 | **2** | **2** | **1** | **1** | **1** | **1** | **1** | **1** |
| 10 | **2** | **1** | **1** | **2** | **2** | **2** | **1** | **1** |
| 11 | **2** | **2** | **2** | **1** | **1** | **1** | **2** | **2** |
| 12 | **1** | **1** | **1** | **2** | **2** | **2** | **2** | **2** |
| 13 | **2** | **1** | **1** | **1** | **1** | **1** | **1** | **1** |
| 14 | **1** | **1** | **1** | **1** | **1** | **1** | **1** | **1** |
| 15 | **2** | **1** | **1** | **1** | **1** | **1** | **1** | **1** |
| 16 | **2** | **2** | **1** | **1** | **1** | **1** | **1** | **1** |
| 17 | **1** | **2** | **2** | **2** | **2** | **2** | **2** | **2** |
| 18 | **2** | **2** | **2** | **2** | **2** | **2** | **2** | **2** |
| 19 | **2** | **1** | **1** | **1** | **1** | **1** | **1** | **1** |
| 20 | 2 | 1 | 1 | 1 | 1 | 1 | 1 | 1 |
| 21 | **2** | **2** | **2** | **1** | **1** | **1** | **1** | **1** |
| 22 | **1** | **2** | **2** | **2** | **2** | **2** | **2** | **2** |
| 23 | **2** | **1** | **1** | **2** | **2** | **2** | **1** | **1** |
| 24 | **2** | **1** | **1** | **2** | **2** | **1** | **1** | **1** |
| 25 | **2** | **2** | **2** | **2** | **2** | **2** | **2** | **2** |
| 26 | **2** | **2** | **2** | **1** | **1** | **1** | **1** | **1** |
| 27 | **2** | **2** | **2** | **2** | **2** | **2** | **2** | **2** |
| 28 | **2** | **2** | **1** | **1** | **2** | **2** | **2** | **2** |
| 29 | **2** | **2** | **2** | **1** | **1** | **2** | **2** | **2** |
| 30 | **2** | **2** | **2** | **1** | **1** | **1** | **1** | **1** |
| 31 | **1** | **2** | **2** | **2** | **2** | **2** | **2** | **2** |
| 32 | **2** | **2** | **2** | **2** | **2** | **2** | **2** | **2** |
| 33 | **2** | **2** | **2** | **1** | **1** | **1** | **1** | **1** |
| 34 | **2** | **2** | **2** | **1** | **1** | **2** | **1** | **2** |
| 35 | **2** | **1** | **1** | **1** | **1** | **1** | **2** | **1** |
| 36 | **2** | **2** | **2** | **2** | **2** | **2** | **2** | **2** |
| 37 | **1** | **2** | **2** | **2** | **2** | **2** | **2** | **2** |
| 38 | **2** | **1** | **1** | **1** | **1** | **1** | **1** | **1** |
| 39 | **2** | **2** | **1** | **1** | **1** | **1** | **1** | **1** |
| 40 | **2** | **1** | **1** | **1** | **1** | **1** | **1** | **1** |
| 41 | **2** | **2** | **2** | **1** | **2** | **2** | **2** | **2** |
| 42 | **2** | **2** | **2** | **1** | **1** | **1** | **1** | **1** |
| 43 | **2** | **2** | **2** | **2** | **2** | **2** | **2** | **2** |
| 44 | **1** | **2** | **2** | **2** | **2** | **2** | **2** | **2** |
| 45 | **2** | **1** | **1** | **2** | **2** | **2** | **2** | **2** |
| 46 | **2** | **1** | **1** | **1** | **1** | **1** | **1** | **1** |
| 47 | **2** | **1** | **1** | **1** | **1** | **1** | **1** | **1** |
| 48 | **2** | **2** | **2** | **1** | **1** | **1** | **1** | **1** |
| 49 | **2** | **2** | **2** | **2** | **2** | **2** | **2** | **2** |
| 50 | **2** | **2** | **2** | **1** | **1** | **1** | **1** | **1** |
| 51 | **1** | **1** | **1** | **1** | **1** | **1** | **1** | **1** |
| 52 | **2** | **2** | **2** | **1** | **2** | **2** | **2** | **2** |
| 53 | **1** | **2** | **1** | **1** | **1** | **1** | **1** | **1** |
| 54 | **2** | **2** | **2** | **1** | **2** | **2** | **2** | **2** |
| 55 | **2** | **2** | **2** | **1** | **1** | **2** | **2** | **2** |
| 56 | **1** | **2** | **2** | **2** | **2** | **2** | **2** | **2** |
| 57 | **2** | **2** | **2** | **2** | **2** | **2** | **2** | **2** |
| 58 | **2** | **2** | **2** | **1** | **1** | **2** | **2** | **2** |
| 59 | **1** | **2** | **2** | **2** | **2** | **2** | **2** | **2** |
| 60 | **2** | **2** | **2** | **1** | **1** | **1** | **1** | **1** |
| 61 | **2** | **1** | **1** | **1** | **1** | **1** | **1** | **1** |
| 62 | **1** | **2** | **2** | **1** | **2** | **2** | **2** | **2** |
| 63 | **2** | **1** | **1** | **1** | **2** | **2** | **1** | **1** |
| 64 | **2** | **1** | **1** | **1** | **1** | **1** | **1** | **1** |
| 65 | **2** | **2** | **1** | **1** | **2** | **2** | **2** | **2** |
| 66 | **1** | **2** | **2** | **2** | **2** | **2** | **2** | **2** |
| 67 | **1** | **2** | **2** | **1** | **2** | **2** | **2** | **2** |
| 68 | **2** | **2** | **2** | **2** | **2** | **2** | **2** | **2** |
| 69 | **2** | **2** | **2** | **2** | **2** | **2** | **2** | **2** |
| 70 | **1** | **1** | **1** | **2** | **2** | **2** | **2** | **2** |
| 71 | **2** | **2** | **2** | **2** | **2** | **2** | **2** | **2** |
| 72 | **2** | **1** | **1** | **2** | **2** | **2** | **2** | **2** |
| 73 | **2** | **2** | **2** | **2** | **2** | **2** | **2** | **2** |
| 74 | **2** | **2** | **2** | **1** | **1** | **1** | **1** | **1** |
| 75 | **2** | **2** | **2** | **2** | **2** | **2** | **2** | **2** |
| 76 | **2** | **2** | **2** | **1** | **1** | **1** | **1** | **1** |

Supplementary table 2

| **Variables** | **Estimate of S1** | **Estimate of S2** | **P-value** |
| --- | --- | --- | --- |
|  |  |  |  |
|  |  |  |  |
| Age (years) | 33.2 | 35.3 | 0.064 |
| BMI (kg/m^2^) | 27.5 | 29.2 | 0.066 |
| Parity | 1.0 | 1.3 | 0.128 |
| Fasting h | 11.5 | 12.3 | 0.192 |
| Hydration L | 2.3 | 2.1 | 0.533 |
| Hb (g/100mL) | 11.3 | 11.2 | 0.706 |
| Birthweight (g) | 3494 | 3569 | 0.410 |
| Baseline SAP (mmHg) | 135.9 | 134.3 | 0.679 |
