# Supplementary material for: A three minutes supine position test reveals higher risk of spinal anesthesia induced hypotension during cesarean delivery. An observational study
Source: F1000Res. 2018 Jul 9;7:1028. [Version 1] doi: 10.12688/f1000research.15142.1 (PMC6085602; doi:10.12688/f1000research.15142.1)
Supplement: Peer review history

# Peer review: Referee response for version 1

**DOI:** 10.5256/f1000research.16496.r36887

**Published:** 2018-08-13

**Authors:** Julian Stander

**Affiliations:** 1 School of Computing, Electronics and Mathematics, University of Plymouth, Plymouth, UK

I am pleased to have the opportunity to comment on this interesting article that has important implications for clinical practice.  I am a statistician and will restrict my comments almost entirely to the statistical analysis.  

**Comments on the Statistical Analysis**

 I find that the statistical analysis presented to be sound and appropriate. 

 I very much appreciate the use of techniques from functional data analysis to extract understanding from these data.  I was curious as to how the smoothing parameter lambda was selected.  I understand that you have performed a thorough study to document the robustness of the results to the choice of lambda.  I would, however, like to see a very brief discussion about whether lambda could be selected automatically, perhaps working in the Bayesian framework.  Implementing and performing such an analysis is clearly beyond the scope of this paper, and I am not asking for it to be done.

 I did not understand from the paragraph beginning "We used the multivariate paired Hotelling's T^2 test..." on page 4 to what S1 and S2 refer.  I think that S1 and S2 should be defined in the previous paragraph.

 For the test H_0: mu_1 = mu_2 = mu_3, I think that H_1 is not precisely stated.  I would state it simply as H_1: not H_0.  

 I have no objection to you performing Hotelling's T^2 tests.  I was, however, wondering whether there were specific tests for  use with functional data.  Perhaps your tests are specific to functional data.  If they are not, a little discussion of this issue would be helpful.

 On page 6 you state that "The clustering algorithm puts each woman in one of two groups, so to divide them in the best way".  In what way is it best please?  It is not quite clear to me from Figure 2, how you would choose the number of groups/clusters.  I was wondering whether this could be done in some automatic way.  Again, implementing and performing such automation is beyond the scope of this paper, but a brief discussion about it would be helpful and interesting.

 I was left a little perplexed by the paragraph beginning "It was evident that the principal component analysis...".  I think that that paragraph needs to be revised with perhaps some more detail.  There could be a relationship here with the comments above about automating certain choices. Further discussion would be valuable.

 General Comment

 In the Abstract, there should be no comma between "observation" and "can".

 I do not think that the Aims read like aims. I would like to see these re-written and then better referred to later in the article in a way that allows me to see that they have been satisfied.

 I think that it would be helpful if you were to remind us about the meaning of the colours on page 7.

I have read this submission. I believe that I have an appropriate level of expertise to confirm that it is of an acceptable scientific standard.

# Peer review: Referee response for version 1

**DOI:** 10.5256/f1000research.16496.r36886

**Published:** 2018-08-08

**Authors:** Gianpaolo Scalia Tomba

**Affiliations:** 1 Department of Mathematics, University of Rome Tor Vergata, Rome, Italy

An interesting study with potential clinical relevance for the identification of women possibly needing reinforced antihypotensive treatment during spinal anesthesia. The study identifies a group of women with stronger SAP reaction to spinal anesthesia based on pre-anesthesia SAP measurement dynamics. The study uses advanced statistical techniques such as smoothing, PCA and clustering to identify the relevant data patterns. Suppl Fig 2 shows how difficult this task would be without the statistical instruments. In the subsequent analysis, smoothing allows the signal to become clear, PCA to reduce the complexity of data and clustering to define groups. In conclusion, a study with a relevant aim, relevant data and relevant statistical analysis.

 Detailed comments

 In the Discussion, the authors point out that a new independent study will be needed to show the reliability of the findings, a wise comment when interesting patterns are found in one data set... Maybe the authors could also comment on how this group finding could be used for an individual classification method... They hint at a individual classification algorithm, but maybe a possible realization of such an algorithm should be at least sketched, especially for a non-statistical readership.

 Otherwise, just minor details:

- Abstract,Background: "...women with decreasing ...(SAP)..." According to later data, all women eventually had decreasing SAP, the problem being approaching a hypotension limit...
- Abstract, Methods: way -> away
- Abstract, Conclusions: As commented above, here a "simple test" is mentioned, some more details would be useful later on...
- Background, para 1,l2: occur-> occurs
- Methods, para 1, l10: are published -> have been published
- Methods, para 3, l5: by to -> by
- Statistical analyses: Maybe the last sentence (about R) should be first...
- Statistical analyses, para 1, l10: in the rest -> in the rest of the paper
- Statistical analyses, para 2, l2: represented in total -> represented
- Statistical analyses, para 3, l6: the negation of H0 is that at least one is different, not that all are different...)
- Results, para 1, l3: why "with increasing values"?
- Results, para 1, l3: principle -> principal
- Results, para 1, l3: The whole sentence beginning on this line should be reformulated, since it is the clustering of the data (blood pressure difference curves) reduced in dimensionality by principal component analysis that allowed the identification of two or three groups of women.
- Results, Fig 1: it would be better, for comparability, if the two panes had the same y-axis
- Results, para 3: the relevance of the analysis of standard deviations is unclear, also whether these are computed on raw SAP or on first order differences. In the Suppl Fig 4, the group S2 seems clearly lower than S1. Does this mean anything? Apparently, these results lead to the clustering in 3 groups...
- Results, para 4: what are the relations between these 3 groups and S1/S2?
- Are the women in T1+T2 = S1 and T3=S2?
- Results, Comparison...supine/left, para 1, l7: maybe "borderline points" is better than "borderline women"...
- Results, Comparison...supine/left, para 1, l7: maybe Fig 2 (and the related last 3 sentences) should be in the preceding subsection...
- Discussion, para 1, l4: no comma (,) after Kinsella et al.
- Discussion, para 4, l9: requires -> require
- Discussion, para 5, l2: provide -> provides
- Discussion, para 6, l4: Hypotension -> hypotension

I have read this submission. I believe that I have an appropriate level of expertise to confirm that it is of an acceptable scientific standard.

# Peer review: Referee response for version 1

**DOI:** 10.5256/f1000research.16496.r35875

**Published:** 2018-07-23

**Authors:** Kim Ekelund

**Affiliations:** 1 Department of Anesthesia, Juliane Marie Center, Copenhagen University Hospital, Rigshospitalet, Copenhagen, Denmark

The present paper is a fine example of how simple questions can be answered by simple studies. The authors have observed that although a standardized approach is used to treat the spinal caused vasodilation during a cesarean section (CS) some women will still experience hypotension despite prophylactic treatment with phenylephrine. They try to identify subsets of women with different susceptibility to vasodilation by register the hemodynamic response after placing them in a supine position for a few minutes. The hypothesis is that some women may need a more aggressive anti-hypotensive treatment during CS and these women might react more when placed on the back, compressing the inferior caval vein and aorta.

 The paper is very interesting because it presents new clinical relevant knowledge on a frequent challenge. 

 The paper is well written, most of the time easy to understand, but is also at some points cut to tight, and I feel as a reader, I am missing information, and this stays with me, despite several attempts to understand. 

**Abstract**

 The abstract is maybe to condense, (maybe due to restriction from the publisher)… I have added a few comments to make the text easier to read… 

 "Cesarean delivery is performed under spinal anesthesia, and vasodilation is the main cause for a drop in blood pressure. *[During supine position]* The compression of the aorta and inferior vena cava by the gravid uterus is of additional clinical importance. *[The spinal caused]* Hypotension may occur during cesarean delivery even if prophylactic infusion of phenylephrine is practiced. We have tested if a 3 minute supine observation, can identify a subset of women with decreasing systolic arterial pressure (SAP) under spinal anesthesia”

**Background**

 “The compression of the aorta and inferior vena cava by the gravid uterus is known to reduce venous return and this is estimated to be of clinical importance after approximately 20 weeks of pregnancy. Pregnant women may find the supine position uncomfortable and avoid this position close to delivery. During cesarean delivery left lateral tilt is recommended to oppose this effect, and theoretically this should reduce the negative effects on maternal circulation”

 Q: Consider moving this paragraph. The paragraphs before and after is about vasodilation – this is not.

**Methods**

 ”They were asked to assess specifically the extra pain caused by to the arterial cannulation”

 Q: What does this mean?

 “The indications for elective cesarean section were maternal request 43%, previous cesarean delivery 25%, breech presentation 8%, other obstetric reasons 13%, and other non-obstetric maternal or neonatal medical conditions 11%.”

 Q: Why is this important in the present study?

 “Concomitantly, we started a rapid intravenous infusion of saline 0.9 mg/ml (37°C, 10 ml/kg) and a phenylephrine bolus (0.25 µg/kg). This was followed with a phenylephrine infusion (0.25 µg/kg/min)”

- and later in the same column:”

Phenylephrine prophylaxis (25 µg/kg/min i.v.) was started when spinal anesthesia was given. Due to the phenylephrine prophylaxis we expected only a minor decrease in SAP after induction of spinal anesthesia”.

 Q: I read this as identical information. And if so – one of the paragraphs should be deleted.

 “The analyses of spinal induced hypotension were based on the beat-to-beat measurements during the first 5 minutes, guided by inspection of the blood pressure curves. Based on a previously published study we expected a maximum decrease in SAP 3–4 minutes after spinal anesthesia ^3^^,^^7^.”

 Q: I believe this should be moved to the Discussion. And be part of an explanation regarding why “three minutes” were chosen, over two, four, five or…. This is missing.

**Statistical analysis:**

 Q: Although it seems to be a thorough explanation, I do not understand the majority. But I was intensively looking for a description of an algorithm, since this is mentioned later in the paper:

 P6: “The clustering algorithm puts each woman in one of two groups, so to divide them in the best way.W

 P8:  ”The external validity of a classification algorithm based on functional data analysis and clustering, as suggested in this paper,…”

 P8: “The algorithm should also be evaluated using a non-invasive continuous monitoring device.”

 Q: What algorithm are the authors referring to?

**Results**

 I have read and re-read the paper the sections regarding the three clusters. I have a hard time understanding the reasons why this subdivision is necessary and more important clinical relevant.

 Q: The authors compare the three clusters in supine position and after spinal. But in the present paper I am not presented with data from the supine position.

**Figure 4.**

 Q: What are T1, T2 and T3, color-explanation is missing.

 Q: The. Red and black curves in Figure 3b and 4b are identical. Explanation?

 Q: Figure 4 text: “…T3.1”? Explanation of the colors should be added, please.

**Discussion**

 ”A statistically significant difference in mean CO was observed between the two groups of women and the group with lowest SAP had a higher CO. The difference in CO was probably of limited clinical significance but indicates that the difference in the compensation of spinal anesthesia induced vasodilatation is

 the mechanism behind this observation. Women are more dependent on sympathetic tone during pregnancy than in non-pregnant state and the effect of spinal anesthesia is a rapid and prominent

 vasodilatation, leading to a decrease in blood pressure and increase in CO7,16. All women in this study received standard phenylephrine bolus and infusion, and this prophylaxis was effective as the mean SAP was stable.”

 Q: Treat the patient not the picture! How were the women doing? Was this clinical relevant?

 “Principal component analyses have been used for making predictive models. In this.  population it would be interesting to identify patients at risk of severe spinal anesthesia induced hypotension and tailor the

 prophylactic treatment.”

 Q: I would be interesting if the authors would share some ideas on how prevent hypotension – an anti-hypotensive adjusted prophylactic treatment?

 Heart rate variability, time domain analysis, and pulse transit time measures are all mentioned in the discussion, and maybe potentially relevant in this clinical setting.

 Q: However, as a reader, I cannot see the obvious link between these investigations and the three minutes supine position test. I would like to have more information, linking to the right population, and to the results of the present study in the discussion, if this information should be considered.

 Q: Finally, I would have preferred if the authors have been more strict on answering their Aims (P5) in the discussion.

I have read this submission. I believe that I have an appropriate level of expertise to confirm that it is of an acceptable scientific standard.

# Response

**Published:** 2018-07-23

**Authors:** Leiv Arne Rosseland

**Affiliations:** Oslo University Hospital, Norway

 Thank you for the positive review of the manuscript. We will respond to the comments and questions and when we have received more referee assessments a revised manuscript will be prepared, including the editing of repeated phrases and the missing information in figure legends.

**Specific questions/answers:**

 ”They were asked to assess specifically the extra pain caused by to the arterial cannulation”

 Q: What does this mean?

 A: The parturients were informed about that arterial cannulation is painful. They were asked to consider if this extra pain could be accepted before considering giving their consent. In the revised manuscript we will describe this better.

 “The indications for elective cesarean section were maternal request 43%, previous cesarean delivery 25%, breech presentation 8%, other obstetric reasons 13%, and other non-obstetric maternal or neonatal medical conditions 11%.”

 Q: Why is this important in the present study?

 A: The generalizability of the study relies on the sample being representative of the population and the information about the included women and the indication for cesarean is included for this reason.

**Statistical analysis:**

 Q: Although it seems to be a thorough explanation, I do not understand the majority. But I was intensively looking for a description of an algorithm, since this is mentioned later in the paper:

 P6: “The clustering algorithm puts each woman in one of two groups, so to divide them in the best way.W

 P8:  ”The external validity of a classification algorithm based on functional data analysis and clustering, as suggested in this paper,…”

 P8: “The algorithm should also be evaluated using a non-invasive continuous monitoring device.”

 Q: What algorithm are the authors referring to?

 In our paper we have used one after the other, three statistical procedures on our data, three algorithms, implemented as packages in the software language R. First we recognise that the measurements made for each women, though they come as a sequence of measures taken in discrete points, represent a function: measures of the pressure exist at any time point, and must be quite smooth. This means that measures made after each other, cannot change too much. The first algorithm estimates such a function for every women. The data for every women are then represented by such a function. The next procedure is finding the most important components of such function, by calculating the principal components and by using the first two components. At this point we have reduced the dimension of the very much, as every women is described by just two numbers (one per principal component). The third procedure is

 clustering: the women are now assigned to one of two clusters, so that within a cluster they are quite similar and across clusters they are quite different. The three procedures realise our algorithm.

 Results

 I have read and re-read the paper the sections regarding the three clusters. I have a hard time understanding the reasons why this subdivision is necessary and more important clinical relevant.

 Q: The authors compare the three clusters in supine position and after spinal. But in the present paper I am not presented with data from the supine position.

 A: The data from supine and left lateral position in the three clusters are presented in Supplementary Table 3.We agree that the clinical relevance of this information (the three clusters) is not obvious. Without this information one might question why we have chosen only the PCA analysis with two clusters. Was there more information hidden that would be revealed when applying three? Or four? Hence, this is kept for methodological reasons.

 Discussion

 ”A statistically significant difference in mean CO was observed between the two groups of women and the group with lowest SAP had a higher CO. The difference in CO was probably of limited clinical significance but indicates that the difference in the compensation of spinal anesthesia induced vasodilatation is

 the mechanism behind this observation. Women are more dependent on sympathetic tone during pregnancy than in non-pregnant state and the effect of spinal anesthesia is a rapid and prominent

 vasodilatation, leading to a decrease in blood pressure and increase in CO7,16. All women in this study received standard phenylephrine bolus and infusion, and this prophylaxis was effective as the mean SAP was stable.”

 Q: Treat the patient not the picture! How were the women doing? Was this clinical relevant?

 A: On average, the women were well treated with the combination of intra-venous fluid + phenylephrine prophylaxis/treatment. However, some patients will develop hypotension and the idea was to analyze if the simple supine position test could predict hypotension. The study should be regarded as an explorative assay, not a pragmatic clinical study. We believe we have demonstrated both the existence of a specific physiologic effect of supine position and that modern signal analyses may be useful in the development of prediction models. The clinical relevance of this has to be tested and proven in future trials.

 “Principal component analyses have been used for making predictive models. In this. population it would be interesting to identify patients at risk of severe spinal anesthesia induced hypotension and tailor the

 prophylactic treatment.”

 Q: I would be interesting if the authors would share some ideas on how prevent hypotension – an anti-hypotensive adjusted prophylactic treatment?

 A: Patients at risk according to this test (or further developments of the test, or other tests) should be treated state of the art to prevent hypotension. This includes lowering the local anesthetic dose injected spinally, increasing the dose of prophylactic phenylephrine (or at least included phenylephrine prophylaxis), left tilting the operating table adequately, and last but not least, consider using beat-to-beat monitoring, invasive or non-invasive monitor. We will include this information  in the revised discussion.

 Heart rate variability, time domain analysis, and pulse transit time measures are all mentioned in the discussion, and maybe potentially relevant in this clinical setting.

 Q: However, as a reader, I cannot see the obvious link between these investigations and the three minutes supine position test. I would like to have more information, linking to the right population, and to the results of the present study in the discussion, if this information should be considered.

 A: We will like to keep this information in the discussion of different methods used and studied for prevention of spinal induced hypotension. If any of these methods prove sensitive and specific as prediction models, it is clinically relevant in the future. We will revise the discussion to make this clearer.

 Q: Finally, I would have preferred if the authors have been more strict on answering their Aims (P5).

 A: Thank you. We will try to indicate more specifically the answers to the aims in results and discussion.
